# American Pharmaceutical Associat’n

**Published:** 1869-10

**Authors:** 


### Editorial Notes.

***American Pharmaceutical Association.***

THE recent meeting of this Association, in this city, (Sept. 7, 1869), was highly successful and satisfactory to the attendants. Delegates enrolled themselves from almost every portion of the country.

Mr. E. H. Sargent, of this city, was elected President for the ensuing year.

The next meeting will be held on the second Tuesday of September, 1870, in the city of Baltimore.

The most important part of their proceedings was discussion of appropriate legislation.

The following is a synopsis of the proposed law to regulate the practice of pharmacy, and the sale of poison, and to prevent the adulteration of drugs and medicines:

Section 1 requires that all shops kept open for the sale and dispensing of medicines and poisons, shall be under the oversight of a registered pharmacist or assistant pharmacist.

Sections 2, 3 and 4 require that no person shall use or exhibit the title of pharmacist or assistant pharmacist unless registered according to law; that no one can register unless a graduate in pharmacy, a practicing pharmacist or assistant pharmacist; that graduates must be from some college of pharmacy in the United States, or from such institution in a foreign country.

Sections 5 and 6 require the appointment of a Pharmaceutical Board by the Governor of each State, and that this board shall examine all candidates for certificates, and cause all prosecutions, and that all members shall pay ten dollars to the board.

Section 8 requires that all registered pharmacists shall furnish their addresses, for which a fee is to be paid. All changes are to be duly sent to the board.

Section 9 imposes a penalty for any false representations in the procuring of registration, by imprisonment from three to twelve months.

Section 10 imposes a fine of fifty dollars for the offence of selling any drugs or medicines, unless he be a registered pharmacist, to be paid to the board.

Sections 11 and 12 authorize the fining of any pharmacist for refusing to comply with the regulations of the board. This is not to interfere with the acts of any practicing physician in the line of his profession.

Section 13 imposes a restriction on the sale of any medicines or poisons, unless the name of the same be placed on the bottle, and the address of the seller. It is the duty of all persons so selling to have the address of the person purchasing, and his name, with a statement of the objects such medicines or poisons are to be used for.

Sections 14, 15 and 16 prohibit the adulteration of any medicines, and require that all prescriptions shall be kept in a book for five years. For adulteration the fine may be $1,000, and imprisonment may be inflicted.

Pending consideration of these propositions, on motion of Dr. Squibb, of New York, the Association adopted the following series of resolutions:

*Resolved*, That the draft of a law to regulate the practice of pharmacy proposed by the committee of the Association, appointed for that purpose, be accepted and published in the proceedings of the Association, as being one method whereby the objects of this body, in regard to that subject, might be attempted; and that, as a method which embraces many useful details, arranged with great care and labor, it is recorded and published as well adapted to be useful to the legislatures of the different states, whenever they may see fit to respond to the earnest desire and call of this Association, and of the community at large, for enactment upon this subject.

*Resolved*, That the difficulties of constructing a form of law proper to be indorsed and recommended by this Association for general application in all the states, are such that we must be satisfied with enunciating the broad principles which, in our judgment, should direct any and all legislation upon this important subject.

*Resolved*, That we see with alarm and regret the rapid increase in the number of accidents which occur from mistakes and mismanagement in dispensing medicinal substances, and that we earnestly desire to see these casualties checked and controlled.

*Resolved*, That we regard the ignorance and irresponsibility of many who engage in the practice of pharmacy, without the proper qualifications, as the principal cause of such casualties.

*Resolved*, That a proper degree of education and qualification should be secured by law, and that all proper measures for educating and qualifying persons for duties so important, should receive more encouragement and protection from the law than they have hitherto done.

*Resolved*, That the report of the committee, embracing the proposed draft of a law, of the action had in this Association upon that report, and of these resolutions, be printed in pamphlet form; and that ten copies be sent to the governors and speakers of the legislatures of the different states of the American Union.

We regret that an additional resolution was not adopted, reprehending the too common practice of druggists prescribing for the patients of physicians who patronize them.

